# An investigation of the specificity and vividness of autobiographical memories and future events produced in response to disgust-related cues among individuals with eating disorders

**DOI:** 10.1186/s40337-025-01214-0

**Published:** 2025-02-24

**Authors:** Sevgi Bektas, Rowan Haslam, Shannon Hilton, Hubertus Himmerich, Valentina Cardi, Janet Treasure, Johanna Louise Keeler

**Affiliations:** 1 Centre for Research in Eating and Weight Disorders (CREW), Institute of Psychiatry, Psychology and Neuroscience, King’s College London, London, SE5 8AF UK; 2 Department of Psychology, Hacettepe University, Ankara, 06800 Türkiye Turkey; 3 Engineering Science Faculty, University College London, London, UK; 4 South London and Maudsley NHS Foundation Trust, London, SE5 8AB UK; 5 Department of General Psychology, University of Padova, Padova, 35122 Italy

**Keywords:** Eating disorders, Autobiographical memory, Episodic future thinking, Moral disgust, Victimisation, Childhood teasing, Betrayal sensitivity

## Abstract

**Background:**

A deficiency in autobiographical memory functioning could be of relevance to the maintenance of an eating disorder (ED). Past research has found that people with EDs have difficulties in recalling specific details of autobiographical memories (AM) and in imagining future events. Our aim was to investigate AM and episodic future thinking (EFT) in individuals with anorexia nervosa (AN), binge-type eating disorders (Bulimia Nervosa or Binge Eating Disorders; BN/BED), and healthy controls (HCs) using negative cue words relevant to the experience of being disgusted and morally violated.

**Methods:**

Remotely administered computerised versions of the autobiographical memory task (AMT) and the EFT task were used to measure the specificity and vividness of AMs and EFTs. Neutral or negative/moral disgust-relevant cues were used to elicit AMs and EFTs. The relationship between AM specificity and EFT specificity was explored. The predictor role of individual differences in childhood teasing and betrayal sensitivity on the specificity and vividness of AMT and EFTs induced by moral disgust-relevant cues was examined.

**Results:**

Individuals with AN and BN/BED did not have difficulties retrieving specific and vivid details of AMs and imagining future events in both cue conditions. AM specificity predicted EFT specificity in AN and HC groups. Future events primed by neutral cues were rated as more vivid by the control group compared to those induced by negative cues. Participants with EDs who had greater levels of childhood teasing and betrayal sensitivity generated more vivid AMs and EFTs in response to moral disgust-related cues, which was not observed in HCs.

**Conclusions:**

This study did not detect alterations in AMT and EFT characteristics in people with AN or binge-type EDs compared with HCs. The findings were discussed regarding sample characteristics (e.g., illness severity, ethnicity) and methodology (e.g., cue words) in the present study. Individual differences in childhood teasing and betrayal sensitivity may be related to more vivid negative memories and future events, which might increase the salience of past and future victimisation-related events.

**Supplementary Information:**

The online version contains supplementary material available at 10.1186/s40337-025-01214-0.

## Abstract (plain language summary)

The study explored the specificity and vividness of autobiographical memories and episodic future thinking, and the predictive role of childhood teasing and betrayal sensitivity on those characteristics in people with eating disorders. The specificity and vividness of memories and future events were comparable between the three groups: anorexia nervosa, binge-type eating disorders, and healthy controls. Participants with eating disorders who experienced higher levels of childhood teasing and who had a greater perception of betrayal sensitivity generated more vivid autobiographical memories and future events in response to moral disgust-related cues, which was not observed in healthy controls. The failure to replicate previous findings, which reported difficulties in autobiographical memory retrieval and future event generation in eating disorder groups compared to healthy controls, may be due to differences in sociodemographic or clinical characteristics between the recruited samples, or due to methodological differences such as the cue words used in the present study. This study also emphasises the possible contribution of individual differences in childhood teasing and the perception of betrayal which may increase the risk of making adverse experiences more salient in people with eating disorders but not in healthy controls.

**Supplementary Information:**

The online version contains supplementary material available at 10.1186/s40337-025-01214-0.

## Introduction

Eating disorders (EDs), including anorexia nervosa (AN), bulimia nervosa (BN) and binge eating disorder (BED), are generally characterised by body dissatisfaction and inappropriate consumption of food (e.g., restricted food intake, bingeing, and purging behaviours), leading to significant distress and negative secondary effects on physical health [1]. Cognitive, emotional and biological processes are thought to underpin these behaviours.

Autobiographical memory (AM) refers to the storage and retrieval of information related to one’s past personal experiences. Within the brain, AM formation involves interactions between the prefrontal cortex, the amygdala, and the hippocampus [2, 3]. Specific AMs refer to personal memories that are usually highly detailed and vivid and function to develop and maintain a sense of self [4]. A recent meta-analysis by Barry et al. [5] has shown that people with psychiatric diagnoses typically recall fewer specific (g = -0.86) and more general (g = 0.71) memories than people without psychiatric diagnoses. Deficits in AM retrieval and overgeneralisations in memory recall appear to be a transdiagnostic feature of a range of different psychiatric diagnoses; however, it has been noted that the majority of studies sampled participants with major depressive disorder (MDD), schizophrenia, and post-traumatic stress disorder (PTSD), with few studies involving other diagnoses. In this regard, research into the role of autobiographical memory in EDs remains limited.

To date, most evidence in the available literature indicates that people with EDs, particularly AN, recall fewer specific AMs towards general negative (e.g., sorry, guilty, hopeless, worse, angry, hurt, lonely) or disorder-relevant cues (e.g., hunger, fat, judgement, weight, stigma, failure) than controls [6, 7, 8, 9, 10, 11, 12, 13, 14, 15, 16]. Similarly, non-clinical studies have observed an impaired AM specificity in individuals with disordered eating [17, 18]. Given the evident association between AM specificity and changes in mood [19], it is important to note that the majority of findings controlled for the influence of depression and AM specificity was still significant different between groups, with the exception of the study by Keeler et al. [11]. It has also been suggested that deficits in recalling autobiographical memories might be an important risk factor for the development and/or maintenance of an ED by interfering with adaptive emotion regulation, planning, problem solving, social functioning, and the ability to imagine possible future events, termed episodic future thinking (EFT) [11, 17, 18]. Hence, more research is needed to ascertain what and how characteristics of AM (i.e., specificity and vividness) underpin eating pathology and body image concerns as well as wider identity processes and perceptions of the future as suggested in a recent review [20].

Williams and colleagues [21] proposed three mechanisms to explain the reduced AM specificity in individuals with depression: ‘capture and rumination’ (CaR), ‘functional avoidance’ (FA), and ‘reduced executive control’ (X). Although the CaR-FA-X was not originally developed for eating disorders, these mechanisms may be applicable to understand why people with EDs show a marked deficit in recalling specific memories. First, ruminative thinking may impede access to specific memories when engaging in AM search process due to the salience and high accessibility of preoccupations in relation to self, food, weight and body image. Second, overgeneral AMs might serve as an implicit emotion regulation strategy for people with EDs, allowing them to avoid re-experiencing strong emotions from the past events. This avoidance may lead to the recall of AMs with less vivid and specific details, as individuals may find it easier to cope with less information. Third, deficits in executive control (e.g. decision-making, cognitive flexibility, planning, organisation, self-control) in people with ED might interfere with the retrieval of specific AMs because of an inability to retain information in mind and ignore irrelevant information. In eating disorders, such as AN, it is likely that aspects of the illness such as low body weight and associated structural and functional changes in the brain [22] may also contribute to problems with retrieving specific details of AMs.

There is a growing body of evidence supporting the potential clinical relevance of the emotions of disgust and self-disgust in EDs. People with EDs have greater levels of disgust towards food, certain parts of the body, or the self that are perceived as misaligning with internalised personal, social, or moral norms [23, 24]. They also report a greater number of negative life experiences ranging from general stressful events to specific food/weight-related or social or interpersonal stressors that violate social norms or moral codes (e.g., teasing, bullying, betrayal) [25], which can induce self-disgust. Based on the functional avoidance element of the CaR-FA-X model, negative events in the amygdala are more likely to be encoded and retrieved with less detail and vividness because of their potency in triggering aversive emotions [26], for example anger and disgust. Such avoidance processes may distort self-perception and influence future expectations. This pattern may be more evident in people with binge spectrum EDs (e.g., BN or BED) who have been found to have an increased level of familial adversity across the life span [27, 28].

von Spreckelsen and colleagues have recently conducted studies to examine the presence of disgust-induced avoidant AM processing in non-clinical female samples, particularly those who appraise their appearance as repulsive, so-called “high repulsive body image”. Women with high repulsive body image recalled a significantly higher proportion of AMs that involved negative appraisals of their own bodies as disgusting [29], and reported a greater level of disgust in response to their body-related AMs [29, 30]. These findings failed to support the reduced AM specificity towards body/weight-related cue words in the group with high repulsive body image [30, 31]. In contrast to the predictions of the CaR-FA-X model, which suggests decreased specificity for negative memories, the findings indicate that these participants may be more biased toward recalling self-disgust-related memories. However, to the best of our knowledge, no study to date has directly examined the relationship between the recall of self- or moral-disgust-related memories and adverse childhood experiences (e.g., bullying, teasing, betrayal).

The hypotheses were as follows: (1) Participants with EDs would recall significantly less specific AMs and less specific future events compared to HCs. The specificity of recalled AMs and constructed future events would vary depending on the valence of the cue (negative/moral disgust-relevant, or neutral) in the clinical group; and following the Constructive Episodic Simulation hypothesis [32], (2) the ability to produce specific details of AMs would predict the ability to produce specific details of EFTs in all three groups. As an exploratory objective, we also aimed to examine the association between self-reported childhood teasing and betrayal sensitivity and the specificity and vividness of AMs and future events induced by negative/moral disgust-relevant cues.

## Methods

### Participants

This study used a cross-sectional between-group design. Participants with AN (*n* = 43), BN (*n* = 12) or BED (*n* = 23) were recruited from the South London and Maudsley NHS Trust (SLaM) and recruitment websites (e.g., BEAT). The HC (*n* = 36) group was recruited via email research circulars at King’s College London (KCL) and social media platforms (e.g., LinkedIn, Twitter). To be eligible for the study, participants had to meet the following criteria: a current diagnosis of ED (for the ED group) or no current ED (for HCs), fluency in English and access to a computer with a stable internet connection. Diagnoses of EDs in the clinical groups were verbally confirmed by participants during a pre-study phone call where a clinical history was taken. Diagnoses were ascertained using Eating Disorder Diagnostic Scale (EDDS) [33, 34] for the screening diagnoses of AN, BN, and BED. All participants were also required to have no history of, or current post-traumatic stress disorder, substance abuse, psychotic disorders, or neurological disorders, as these psychiatric and neurological conditions are associated with difficulties with AM retrieval [35] A total of 114 participants met al.l criteria for inclusion and completed the study.

A power analysis using G x Power 3.1, based on the effect size reported in a previous study that compared AM retrieval and EFT in individuals with acute AN, recovered AN, and unaffected controls [11], indicated that the current sample allowed for detection of a medium effect size with 95% power and a significance level of 5%. Sample characteristics and between-group comparisons are presented in Tables 1 and 2.

### Measures

#### Demographics and clinical variables

The demographic questionnaire measured the following variables: age, gender, ethnicity, years of education, highest level of education, living and employment status, dietary lifestyle, and medication history. Clinical characteristics collected included the type of current diagnosis of ED, illness duration (how long ago they first experienced symptoms of an ED, and how long ago they were first diagnosed with an ED). Self-reported weight and height details were also recorded, which were used to calculate body mass index (BMI) in kg/m^2^.

#### Eating disorder psychopathology and characteristics

*Eating Disorder Examination Questionnaire (EDE-Q 6.0)* [36, 37]. The EDE-Q is a 28-item questionnaire assessing ED psychopathology severity with a global score and four subscales: dietary restraint, shape concern, weight concern, and eating concern. Scores range from 0 to 6, with higher scores indicating greater severity. The Cronbach’s alpha of the global score in this study was 0.97.

*Binge Eating Scale (BES)* [38]. The BES is a 16-item self-report scale that assesses binge eating severity. The total score ranges from 0 to 46, and the higher the score, the more severe the binge eating problems. The Cronbach’s alpha in this study was 0.95.

#### Comorbidities

Participants were asked to indicate if they had received a diagnosis of the following comorbid psychiatric disorders: affective disorder, anxiety disorder, obsessive-compulsive disorder (OCD), and attention deficit hyperactivity disorder (ADHD), and autism spectrum disorder (ASD).

*Depression*,* Anxiety and Stress Scale-21 (DASS-21)* [39]. The DASS is a 21-item self-report questionnaire measuring symptoms of depression, anxiety and stress over the previous week using a series of statements and responses ranging from 0 to 3. The Cronbach’s alpha in this study was 0.94.

#### Disgust-related variables

*Disgust Scale Revised (DS-R)* [40] The DS-R is a 25-item questionnaire measuring individuals’ disgust propensity across three disgust domains: core disgust, animal-reminder disgust, and contamination. The Cronbach’s alpha in this study was 0.89.

*Self-Disgust Scale Revised (SDS-R)* [41]. The SDS-R is a 22-item questionnaire measuring self-disgust. It provides two subscale scores (physical self-disgust and behavioural self-disgust), and a total score related to general self-disgust. A total score is calculated with 15 items, with higher scores indicating greater levels of self-disgust sensitivity. The Cronbach’s alpha in this study was 0.96.

#### Other psychological variables

*Teasing Questionnaire – Revised (TQ-R)* [42]. The TQ-R is a 29-item self-report measure of recalled childhood teasing related to performance, academic issues, social behaviour, family background, and appearance. Higher scores indicate a greater number of teasing experiences. The Cronbach’s alpha in this study was 0.93.

*Perception of Betrayal Scale (POBS)* [43]. The POBS is a 27-item questionnaire which assess the impact of betrayal on different aspects such as self-perception, interpersonal relationships, and behaviour. Higher scores indicate greater betrayal sensitivity. The Cronbach’s alpha in this study was 0.98.

*Moral Orientation Guilt Scale (MOGS)* [44]. The MOGS, a 17-item scale measures individuals’ propensity to experience different types of guilt (moral norm violation, moral dirtiness, empathy, and harm) linked to deontological values. The Cronbach’s in this study was 0.89.

#### Sleep quality and sleepiness

Sleep quality and sleepiness have been found to interfere with autobiographical memory retrieval [45], which may be a potential confounding factor. Hence, sleep quality and sleepiness were measured using the Epworth Sleepiness Scale (ESS) [46], which is a widely used 8-item scale from 0 (no chance of dozing) to 3 (high chance of dozing). Participants were also asked to specify how long they slept in hours over the previous three nights, which was used to compute an average. The Cronbach’s alpha for the ESS in this study was 0.79.

#### Autobiographical memory and episodic future thinking

*Autobiographical Memory Test (AMT)* [47]. The AMT involves the presentation of a series of cue words, to which participants are asked to generate a description of a memory. For EFT, a variant of the AMT (the EFT task; EFT-T) was used, which has an identical procedure but with the instruction to simulate a hypothetical or likely event rather than recall a memory [48]. Participants completed computerised written versions of the AMT and EFT-T where they were given two minutes to generate a specific event they personally experienced in the past (AM) or might experience in the future (EFT). Instructions at the beginning of the task informed participants that the memory or future event should be a specific, personal experience that had lasted, or would last, no longer than one day. The future event should be a hypothetical or likely personal event inspired by or directly related to the cue word. Participants were instructed to consider as many details of the memory/future event as possible (i.e., what is being done, who they are with, feelings and emotions). Participants were also instructed that a different memory should be used for each cue, although no restrictions on the time frame were made. Participants were given two examples of responses to word cues. See Table S1 for instructions with two examples for both AMT and EFT-T.

The words shown to participants consisted of 12 cues, including six neutral and six negative cues per task (12 trials per participant). The neutral cues (book, table, chair, pen, window, and room) were selected from the list utilised by Keeler et al. [11]. For negative cues, words were chosen that described a violation of one’s personal or socio-moral values or norms and most intensely trigger feelings of disgust [49]. The authors collaborated with a Patient and Public Involvement (PPI) group of native speakers with lived experience of an ED (*n* = 6), and with healthy controls (*n* = 9), to rate a list of negative cue words (betrayal, teasing, mistrust, shunned, exclusion, bullying, mocking, disloyalty, shame, and let-down) in terms of to what extent each word triggered feelings of disgust towards themselves, others, or the situation, and violated personal, socio-moral values or norms, from 0 (not at all) to 5 (extremely) (see Table S2). The PPI group was also asked to generate cues or words relevant to their experience of being disgusted and morally violated. The frequency of ratings was analysed, and six words (shame, mistrust, disloyalty, exclusion, let down, and bullying) that elicited the highest frequency ratings of disgust and violation were chosen for inclusion in the task. These words were then used in online tasks, where participants received a 30-second rest break between trials. The primary dependent variable was the specificity of AMs and EFTs.

*Specificity Coding.* AMs and EFTs were coded independently by two researchers (S.B. and R.H.). Both researchers were blind to participants’ diagnostic status. AMs and EFTs were coded as ‘specific’ if they occurred or could occur within 24 h and were located in a time and place. AMs and EFTs referring to a particular time period that lasted longer than 24 h were coded as `extended`, and those that have occurred repeatedly were coded as `categorical`. AMs and EFTs that simply referred to objects, places, or people without a context were coded as `semantic.` Decisions not to respond to a cue word or failures to retrieve specific memories to cues within the time limit were coded as `omissions.` Additionally, the two raters could categorise AMs and EFTs into another general memory category, `off-task` if they are violating the task instructions. Descriptive characteristics of each of memory/future event categories: (1) specific, (2) extended, (3) categorical, (4) semantic (5) off-task per cue type and group were given in Table S3. The proportion of specific memories/future events, adjusted for omissions, was calculated for each group and each type of cue word. For example, in response to negative cues (*n* = 3), if a participant retrieved one specific memory and made one omission, then the proportion would be calculated using the following Eq. 1/ (3 − 1) =, which would be 0.50 (50%).

*Secondary Dependent Variables*. Additional dependent variables were the participants’ ratings of how vivid the memory/future event was and how disgusted the participants were by the generated memory/future event. For these variables, participants were asked to rate their answers on a series of 7-point Likert scales (1 = not at all, 7 = extremely). Per memory/future event, participants were asked what the source(s) (who or what) of the disgust feelings was from the following options: me; family; friends; peers; strangers; the event itself; there was no feeling of disgust; or other. If their answer was “other” participants were directed to specify the source.

### Procedure

Informed consent was obtained from all participants using an approved participant information sheet and consent form, and the study received Health Research Authority (HRA) and Health and Care Research Wales (HCRW) approvals from the London Bridge NHS Research Ethics Committee (Reference: 18/LO/0121). Participants were given £10 for their time.

This study used the online platforms Qualtrics (www.qualtrics.com) and Gorilla [50] to create and host tasks. Eligible participants received a phone call or email from a researcher (S.B.) where they were introduced to the study and instructed on how to optimise the study environment for completing the tasks, such as turning off their mobile phones, ensuring they were in a quiet space with minimal distractions, and putting the browser in full-screen mode.

Researchers were able to monitor participants’ progress in the study through the web interface. The study took approximately 1.5 h. A researcher was available during the study session over email and telephone, in case participants needed the task instructions to be explained in more detail. Prior to the first session for the AMT and EFT-T, participants were sent a link to complete a battery of questionnaires. At the end of the AMT and EFT-T, a short positive mood induction (3 min) of relaxing music was administered.

### Statistical analysis

All analyses were conducted using The Statistical Package for the Social Sciences (SPSS) [51] Pearson Chi-square test statistics (for categorical data) and ANOVAs (for continuous data) with post-hoc Tukey tests (for multiple comparisons) were conducted to compare socio-demographic, clinical and psychometric variables among groups. The Tukey test was used for multiple comparisons.

Individual analyses of covariance (ANCOVA) models were run using the AMT and EFT-T outcomes as dependent variables, within a 2 (cue valence: negative and neutral) x 3 (group: control, AN, and BN/BED) model, with age and ethnicity entered as covariates. Sensitivity analyses (TablesS4 and S5) were run in order to examine whether removing Asian participants from the sample (*n* = 20) and controlling for DASS-Depression scores would alter the results.

The analysis of disgust ratings to future events was conducted using nonparametric methods (Quade’s ANCOVA) per cue type, as the data did not meet the assumptions required for parametric testing.

To assess the relationship between AMT specificity and EFT specificity, a linear regression analysis using the “enter” procedure was conducted separately for each group with age and ethnicity as covariates, EFT specificity (the proportion of specific future events) as the dependent variable and AM specificity (the proportion of specific autobiographical memories) as a predictor. Regression models also examined the relationship of childhood teasing and betrayal sensitivity with AMT and EFT task outcomes (specificity and vividness in response to negative cues). For the second regression analysis, we combined both AN and BN/BED groups into a single category to improve the statistical power of the regression analysis. The effects of control variables (age and ethnicity [Caucasian vs. non-Caucasian]) were adjusted for by entering them together with childhood teasing or betrayal sensitivity in the same step for each model. The *p* < 0.05 threshold of significance was utilised for all analyses, and effect sizes are reported in the form of Eta Squared (η2) and standardized *ß* in the case of regression models. All post-hoc group-comparisons were Bonferroni corrected.

## Results

### Socio-demographic and clinical characteristics

The descriptive statistics and the results of the groups comparisons are reported in Table 1.

All groups were comparable in terms of gender, years of education, and duration of diagnosis and symptom, however, age and ethnicity differed between groups. There was greater ethnic diversity in the control and BN/BED group compared with AN. The BN/BED group was older than the control group. As expected, self-reported BMI significantly differed between groups, with the BN/BED group reporting a higher BMI than controls, and the AN group reporting a lower BMI than controls. Thirteen participants (30%) in the AN group had a BMI of ≥ 18.5 kg/m^2^. The proportion of participants with AN and binge-type ED currently under treatment (e.g., inpatient, outpatient, or private care) were 67.4% and 29.4%, respectively.

**Table 1:** Comparison of socio-demographic and clinical characteristics between HC, AN and mixed group of BN and BED

	HC (*n* = 36)	AN (*n* = 43)	BN/BED (*n* = 35)	F-value (df) or X^2^ (df)	*p* value (Partial Eta Squared, η2 or Phi, φ)	*p* value for post hoc comparisons
Age, years M ± SD	24.69 ± 6.01	28.53 ± 6.97	31.83 ± 9.55	7. 85 (2, 111)	< 0.001 ** (0.12)	AN vs. HC = 0.069
						BN/BED vs. HC < 0.001 **
						AN vs. BN/BED = 0.142
Gender, n (%)				2.77 (4)	0.60 (0.16)	
Female	33 (91.7%)	40 (93.0%)	34 (97.1%)			
Male	3 (8.3%)	2 (4.7%)	1 (2.9%)			
Non-binary	-	1 (2.3%)	-			
BMI, kg/m^2^ M ± SD	21.40 ± 2.47	16.98 ± 2.22	34.52 ± 12.62	59.23 (2, 111)	< 0.001 ** (0.52)	AN vs. HC = 0.022 *
						BN/BED vs. HC < 0.001 **
						AN vs. BN/BED < 0.001 **
Ethnicity, n (%)				33.08 (10)	< 0.001 ** (0.54)	
White	17 (47.2%)	40 (93.0%)	26 (74.3%)			
Black or African	-	-	1 (2.9%)			
Asian	13 (36.1%)	2 (4.7%)	5 (14.3%)			
Hispanic/Latino	-	-	1 (2.9%)			
Mixed Race	1 (2.8%)	1 (2.3%)	2 (5.7%)			
Other	5 (13.9%)	-	-			
Years of Education, M ± SD	17.15 ± 2.28	17.51 ± 2.35	17.60 ± 2.76	0.30 (2, 95)	0.75 (0.05)	
Diagnosis Duration (years, M ± SD)	-	10.00 (7.75)	8.62 (9.59)	2.03 (74)	0.16 (0.01)	
Symptom Duration (years, M ± SD)		12.49 (8.27)	16.68 (11.04)	3.29 (74)	0.07 (0.05)	
Comorbidity, n (%)						
Obsessive Compulsive Disorder	-	5 (11.6%)	1 (2.9%)			
Affective Disorder	-	17 (39.5%)	11 (31.4%)			
Anxiety Disorder	-	15 (34.9%)	14 (40%)			
Attention Deficit Hyperactivity Disorder	-	1 (2.3%)	4 (11.4%)			
Autism Spectrum Disorder	-	4 (9.3%)	2 (5.7%)			
Current Treatment, n (%)				10.97 (1)	< 0.001 ** (-0.38)	
Treatment	-	29 (67.4%)	10 (29.4%)			
No Treatment	-	14 (32.6%)	24 (70.6%)			

* Significant at the *p* < 0.05 threshold, ** Significant at the *p* < 0.001 threshold. All post-hoc analyses were conducted with the Tukey method. Abbreviations: AN = Anorexia Nervosa; BMI = Body Mass Index; kg/m^2^ = kilogram per square metre; BN/BED = Bulimia Nervosa/Binge Eating Disorder; HC = Healthy Controls; M = Mean; SD = Standard Deviation

### Self-report ratings of psychopathology and individual differences

The descriptive statistics and results of the group comparisons are reported in Table 2.

ED psychopathology differed between groups, with both the AN and BN/BED groups scoring higher in EDE-Q global scores and all subscales compared with HCs. Binge eating was greater in BN/BED and AN compared with HC, but also greater in BN/BED compared with AN. The DASS scores differed between groups. Participants with BN/BED reported higher sleepiness scores compared with those with AN. However, the average duration of sleep over the previous three nights did not differ between groups.

The disgust sensitivity scores were similar between groups. However, levels of self-disgust sensitivity, childhood teasing, betrayal sensitivity, and three MOGS subscales: moral norm violation, empathy, and moral dirtiness differed between groups. All scores were significantly higher in the ED groups compared to HCs.

**Table 2:** Comparison of self-report ratings between HC, AN, and mixed group of BN and BED

	HC (*n* = 36)	AN (*n* = 43)	BN/BED (*n* = 35)	F-value (df) or X^2^ (df)	*p* value (Partial Eta Squared, η2)	Post-hoc comparisons (*p*-value)		
						AN vs. HC	BN/BED vs. HC	AN vs. BN/BED
EDE-Q, M ± SD								
Global	0.68 ± 0.62	3.72 ± 1.24	3.95 ± 1.30	100.40 (2, 111)	< 0.001 ** (0.64)	< 0.001 **	< 0.001 **	0.630
Restraint	0.44 ± 0.69	3.62 ± 1.61	3.01 ± 2.00	45.43 (2, 111)	< 0.001 ** (0.45)	0.001 **	< 0.001 **	0.190
Eating Concern	0.29 ± 0.34	3.12 ± 1.54	3.51 ± 1.64	64.49 (2, 111)	< 0.001 ** (0.54)	< 0.001 **	< 0.001 **	0.390
Shape Concern	1.06 ± 0.97	4.26 ± 1.27	4.74 ± 1.28	103. 61 (2, 111)	< 0.001 ** (0.65)	0.001 **	< 0.001 **	0.180
Weight Concern	0.93 ± 0.92	3.88 ± 1.41	4.55 ± 1.26	89.36 (2, 111)	< 0.001 ** (0.62)	< 0.001 **	< 0.001 **	0.050
BES, M ± SD	6.50 ± 4.12	17.45 ± 9.39	32.65 ± 8.10	104.10 (2, 111)	< 0.001 ** (0.65)	< 0.001 **	< 0.001 **	< 0.001 **
DASS, M ± SD								
Depression	7.00 ± 8.55	19.86 ± 11.97	20.80 ± 10.33	19.85 (2, 111)	< 0.001 ** (0.26)	< 0.001 **	< 0.001 **	0.918
Anxiety	6.61 ± 6.81	11.91 ± 6.94	11.20 ± 20.32	5.26 (2, 111)	0.007 * (0.09)	0.008 *	0.035 *	0.918
Stress	11.28 ± 8.92	22.05 ± 8.94	20.32 ± 11.16	13.47 (2, 111)	< 0.001 ** (0.20)	< 0.001 **	< 0.001 **	0.713
DS-R, M ± SD	2.25 ± 0.63	2.29 ± 0.73	2.38 ± 0.81	0.29 (2, 111)	0.747 (0.00)	-	-	-
SDS Total, M ± SD	31.72 ± 12.49	69.98 ± 15.96	72.80 ± 16.79	83.28 (2, 111)	< 0.001 ** (0.60)	< 0.001 **	< 0.001 **	0.700
TQ-R, M ± SD	15.44 ± 11.65	26.14 ± 16.69	31.00 ± 22.34	7.54 (2, 111)	< 0.001 ** (0.12)	0.020 *	< 0.001 **	0.440
POBS, M ± SD	44.64 ± 33.91	88.48 ± 38.82	91.98 ± 44.21	16.66 (2, 111)	< 0.001 ** (0.23)	< 0.001 **	< 0.001 **	0.920
MOGS, M ± SD								
Moral Norm Violation	17.83 ± 3.85	20.70 ± 5.47	21.29 ± 5.19	5.11 (2, 111)	0.008 * (0.08)	0.030 *	0.010 *	0.860
Empathy	15.64 ± 3.51	18.02 ± 4.47	18.43 ± 4.22	4.89 (2, 111)	0.009 * (0.08)	0.030 *	< 0.01 *	0.900
Moral Dirtiness	6.33 ± 2.23	10.26 ± 2.98	10.43 ± 3.05	25.39 (2,111)	< 0.001 ** (0.31)	< 0.001 **	< 0.001 **	0.960
Harm	13.11 ± 2.01	13.35 ± 1.91	12.97 ± 2.70	0.29 (2, 111)	0.747 (0.01)	-	-	-
Average sleep last 3 days, hours M ± SD	7.23 ± 0.79	6.67 ± 1.24	6.74 ± 1.70	2.10 (2, 111)	0.127 (0.04)	-	-	-
Epworth Sleepiness Score, M ± SD	6.73 ± 3.22	6.34 ± 4.54	8.68 ± 4.66	0.91 (2, 111)	0.041 * (0.06)	0.920	0.130	0.040 *

Notes. * Significant at the *p* < 0.05 threshold, ** Significant at the *p* < 0.001 threshold. All post-hoc analyses were conducted with the Tukey method. Abbreviations: Anorexia Nervosa; BES = Binge Eating Scale; BN/BED = Bulimia Nervosa/Binge Eating Disorder; DASS = Depression Anxiety Stress Scale; DS-R = Disgust Scale – Revised; EDE-Q = Eating Disorder Examination – Questionnaire; HC = Healthy Controls; M = Mean; MOGS = Moral Orientation Guilt Scale; POBS = Perception of Betrayal Scale; SD = Standard Deviation; SDS-R = Self-Disgust Scale – Revised; TQ-R = Teasing Questionnaire – Review; VAS = Visual Analogue Scale

### Autobiographical memory and episodic future thinking

Means and standard deviations of the AMT and EFT-T task outcomes (the proportion of specific autobiographical memories/future events, ratings of vividness and disgust) per cue type between groups are presented in Table 3.

**Table 3:** Mean and standard deviations for dependent variables between groups

	HC		AN		BN/BED	
	Neutral cue words Mean (SD)	Negative cue words Mean (SD)	Neutral cue words Mean (SD)	Negative cue words Mean (SD)	Neutral cue words Mean (SD)	Negative cue words Mean (SD)
Autobiographical Memory Test (AMT)						
Specificity ^a^	0.55 (0.33)	0.53 (0.39)	0.69 (0.33)	0.60 (0.33)	0.69 (0.29)	0.65 (0.35)
Vividness ^b^	5.41 (1.11)	4.94 (1.42)	5.19 (1.14)	5.16 (1.31)	5.61 (1.18)	5.52 (1.26)
Disgust ^b^	2.16 (1.14)	3.12 (1.69)	3.01 (1.51)	4.22 (1.79)	3.08 (1.48)	4.93 (1.48)
Episodic Future Thinking Task (EFT)						
Specificity ^a^	0.61 (0.35)	0.35 (0.34)	0.59 (0.37)	0.39 (0.41)	0.50 (0.37)	0.35 (0.38)
Vividness ^b^	5.05 (1.44)	3.94 (1.35)	4.50 (1.31)	4.62 (1.47)	4.43 (1.29)	4.58 (1.45)
Disgust ^b^	1.40 (0.59)	3.38 (1.64)	2.28 (1.50)	4.09 (1.77)	2.17 (1.59)	4.67 (1.40)

*Notes.*^a^ Researcher rated ^b^ Participant rated. Specificity refers to the proportion of autobiographical memories/future events. Abbreviations: AN = Anorexia Nervosa; BN/BED = Bulimia Nervosa and Binge Eating Disorders; HC = Healthy Controls; SD = Standard Deviation

Table 4 presents the main effects of cue valence, group and cue valence x group for each task-related outcome.

**Table 4:** Effect of cue valence, group, and cue valence x group in ANCOVA models for autobiographical memory test (AMT) and episodic future thinking task (EFT-T) outcomes

Outcome	Cue Valence		Group		Cue Valence x Group	
	**F-value (1**,**109)**	***p*** **value (η2)**	**F-value (2**,**109)**	***p*** **value (η2)**	**F-value (3**,**109)**	***p*** **value (η2)**
*Autobiographical Memory Test*						
Specificity ^a^	0.090	0.765 (0.001)	1.268	0.285 (0.023)	1.623	0.202 (0.029)
Vividness ^b^	0.492	0.485 (0.004)	1.765	0.176 (0.031)	0.384	0.682 (0.007)
Disgust ^b^	7.137	0.009 ** (0.061)	6.881	0.002 ** (0.112)	2.838	0.063 (0.049)
*Episodic Future Thinking Task*						
Specificity ^a^	0.717	0.399 (0.007)	0.295	0.745 (0.005)	0.440	0.645 (0.008)
Vividness ^b^	2.981	0.087 (0.027)	0.008	0.992 (0.000)	6.024	0.003 ** (0.100)
Disgust ^b^	15.259	< 0.001 *** (0.123)	5.776	0.004 ** (0.096)	1.974	0.144 (0.035)

*Notes.* * Significant at the *p* < 0.05 threshold, ** Significant at the *p* < 0.01 threshold. *** Significant at the *p* < 0.001 threshold. ^a^ Researcher rated ^b^ Participant rated. Specificity refers to the proportion of specific autobiographical memories/future events. All analyses were run with age and ethnicity entered as covariates

### Autobiographical memory test

***Specificity ratings.*** No significant group x valence interaction effects, nor main effects of group or valence, were observed for the proportion of specific autobiographical memories.

***Vividness ratings.*** Similarly, there were no significant group x valence interaction effects, nor main effects of group or valence, for the rated vividness of autobiographical memories.

***Disgust ratings.*** There was no group x valence interaction effect for the disgust ratings towards autobiographical memories. However, the effect of valence was significant for disgust feelings to autobiographical memories (F (1, 109) = 7.137; *p* = 0.009; η2 = 0.061), whereby memories in response to negative cues were rated with a greater level of disgust than memories in response to neutral cues (*p* < 0.001; 95% CI [1.049, 1.647]). The effect of group was also significant for disgust feelings to autobiographical memories (F (2, 109) = 6.881; *p* = 0.002; η2 = 0.112). Individuals with BN/BED reported greater levels of disgust to autobiographical memories (*p* = 0.001; 95% CI [0.426, 2.062]) regardless of cue valence, compared to HCs. When controlling for DASS-Depression scores, the cue valence effect for the disgust to memories did not change (F (1, 109) = 5.501; *p* = 0.021; η2 = 0.048) although the group effect for disgust to memories was non-significant (F (2, 109) = 1.142; *p* = 0.323; η2 = 0.021) (see Table S4).

### Episodic future thinking Task

***Specificity ratings***. In the EFT-T, there was no significant group x valence interaction effects for the proportion of specific future events, nor main effects of group or valence.

***Vividness ratings.*** A significant interaction between group and valence emerged for the vividness of future events only (F (3, 109) = 6.024; *p* = 0.003; η2 = 0.100). Post-hoc comparisons revealed that future events induced by negative cues were rated as less vivid (*p* < 0.001, 95% CI [0.511, 1.545]) in HCs compared to those induced by neutral cues. This was non-significant when controlling for DASS-Depression scores (Table S4). However, after controlling for DASS-Depression scores, the effect of valence on the vividness of future events was significant (F (1, 109) = 9.980; *p* = 0.002; η2 = 0.085) (see Table S4), whereby the vividness of future events primed by negative cues was lower than future events primed by neutral cues (*p* < 0.001; 95% CI [1.779, 2.433]).

***Disgust ratings.*** There was no group x valence interaction effect for disgust ratings to EFTs. However, the effect of valence was significant for disgust feelings to future events (F (1, 109) = 15.259; *p* < 0.001; η2 = 0.123), whereby future events induced by negative cues were reported as more disgusting in comparison with those induced by neutral cues in the whole sample (*p* < 0.001; 95% CI [1.779, 2.430]). The effect of group was also significant for disgust feelings to future events (F (2, 109) = 5.776; *p* = 0.04; η2 = 0.096). Both AN (*p* = 0.048; 95% CI [0.004, 1.439]) and BN/BED (*p* = 0.004; 95% CI [0.278, 1.779]) reported higher levels of disgust to future events in comparison with HCs. When controlling for DASS-Depression scores, the cue valence effect for the disgust to future events did not change (F (1, 109) = 11.242; *p* < 0.001; η2 = 0.094) although the group effect on disgust to future events was non-significant (F (2, 109) = 0.422; *p* = 0.657; η2 = 0.008). The Quade’s ANCOVA findings revealed a significant main effect of group on disgust feelings to future events induced by negative cues (F (2, 114) = 3.882, *p* = 0.023). Specifically, individuals with BN/BED reported significantly greater levels of disgust to future events induced by negative cues in comparison with HCs (*p* = 0.018; 95% CI [2.961, 38.703]). No significant difference in disgust to future events was observed for neutral cues among groups (see Table S6).

When removing Asian participants from the sample (*n* = 20), all main results remained the same (Table S5).

### The effect of autobiographical memory specificity on episodic future thinking specificity

Overall, all models significantly predicted EFT-T specificity, both with and without the inclusion of age, ethnicity as regressors, for only AN and HC groups (see Table 5). In HCs, AMT specificity accounted for 12% of variance in EFT-T specificity (F (1,34) = 5.739; *p* = 0.022). In AN, AMT specificity accounted for 29% of variance in EFT-T specificity (F (1, 41) = 17.920; *p* < 0.001). In both groups, the inclusion of age and ethnicity did not significantly contribute to the predictive value of the models (all *p* > 0.05).

**Table 5:** Results of linear regression models investigating the effect of AM specificity (model 1) and control regressors (age and ethnicity; model 2) on the EFT specificity, stratified by group

Model	Adjusted *R*^2^ (SE)	Independent Variable	Unstandardised Beta (SE)	ß	T	*p* value
*HC*						
1	0.119 (0.268)	(Constant)	0.300 (0.090)		3.314	0.002 *
		AMT Specificity	0.347 (0.145)	0.380	2.396	0.022 *
2	0.096 (0.271)	(Constant)	0.099 (0.262)		0.378	0.708 *
		AMT Specificity	0.391 (0.153)	0.429	2.5596	0.015 *
		Age	0.005 (0.008)	0.096	0.565	0.576
		Ethnicity	0.024 (0.023)	0.175	1.021	0.315
*AN*						
1	0.287 (0.284)	(Constant)	0.116 (0.100)		1.158	0.254
		AMT Specificity	0.595 (0.141)	0.551	4.233	< 0.001 **
2	0.261 (0.261)	(Constant)	0.212 (0.242)		0.876	0.386
		AMT Specificity	0.617 (0.147)	0.572	4.209	< 0.001**
		Age	-0.002 (0.007)	-0.043	-0.043	0.761
		Ethnicity	-0.042 (0.056)	-0.108	-0.752	0.457
*BN/BED*						
1	0.037 (0.334)	(Constant)	0.228 (0.150)		1.524	0.137
		AMT Specificity	0.311(0.204)	0.256	1.524	0.137
2	0.060 (0.330)	(Constant)	0.573 (0.257)		2.232	0.033
		AMT Specificity	0.278 (0.208)	0.229	1.337	0.191
		Age	-0.007 (0.032)	-0.212	-1.271	0.213
		Ethnicity	-0.049 (0.228)	-0.196	-1.145	0.261

*Notes.* * Significant at *p* < 0.05, **Significant at *p* < 0.001. Specificity refers to the proportion of specific memories/future events. Abbreviations: AMT = Autobiographical Memory Test; AN = Anorexia Nervosa; BN/BED = Bulimia Nervosa/Binge Eating Disorder; HC = Healthy Control; SE standard error; ß standardised beta

### The association between victimisation experiences and specificity and vividness of AMTs and EFTs induced by negative cues

Overall, all models evaluating the effect of childhood teasing and betrayal sensitivity on the specificity of AM and EFT were non-significant in both ED and HC groups. However, overall models for vividness ratings were significant in the ED group. Victimisation experiences were significantly associated with both AM vividness (β = 0.298, *p* = 0.006 for TQ-R and β = 0.377, *p* < 0.001 for POBS) and EFT vividness (β = 0.271, *p* = 0.018 for TQ-R and β = 0.483, *p* < 0.001 for POBS), indicating that higher experiences of victimisation were associated with more vivid AMs and EFTs. These significant predictions were observed in the ED sample but not in the HC (see Table S7). The results of regression analyses for the ED sample are presented in Table 6.

**Table 6:** Results of linear regression models investigating the effect of TQ-R and POBS on AMT and EFT-T outcomes in response to negative cues in the ED sample (*n* = 78)

Tested Model	Dependent Variable	Adjusted *R*^2^ (SE)	F-value (df = 3,74)	*p*-value for overall model	Independent Variable	Unstandardised Beta (SE)	ß	t	*p*-value for regressor
***Autobiographical Memory Task***									
Model 1	Specificity	-0.014 (0.340)	0.650	0.585	**(Constant)**	0.684 (0.172)		3.966	< 0.001 **
					TQ-R	-0.072 (0.058)	-0.142	-1.236	0.220
					Age	0.001 (0.005)	0.029	0.251	0.803
					Ethnicity	-0.019 (0.034)	-0.063	-0.545	0.587
Model 2	Vividness	0.142 (1.195)	5.238	0.002*	**(Constant)**	4.718 (0.605)		7.795	< 0.001 **
					TQ-R	0.574 (0.204)	0.298	2.817	0.006 *
					Age	0.016 (0.016)	0.102	0.958	0.341
					Ethnicity	-0.303 (0.121)	-0.267	-2.511	0.014 *
Model 3	Specificity	-0.029 (0.343)	0.278	0.841	**(Constant)**	0.652 (0.179)		3.643	< 0.001 **
					POBS	-0.017 (0.026)	-0.075	-0.646	0.520
					Age	0.002 (0.005)	0.042	0.362	0.719
					Ethnicity	-0.018 (0.035)	-0.061	-0.521	0.604
Model 4	Vividness	0.197 (1.156)	7.304	< 0.001 **	**(Constant)**	4.383 (0.603)		7.262	< 0.001 **
					POBS	0.319 (0.087)	0.377	3.687	< 0.001 **
					Age	0.010 (0.016)	0.066	0.641	0.524
					Ethnicity	-0.305 (0.117)	-0.268	-2.607	0.011 *
***Episodic Future Thinking Task***									
Model 5	Specificity	-0.014 (0.394)	0.647	0.587	**(Constant)**	0.557 (0.200)		2.788	0.007 *
					TQ-R	0.005 (0.067)	0.008	0.073	0.942
					Age	-0.004 (0.005)	-0.085	-0.730	0.468
					Ethnicity	-0.050 (0.040)	-0.144	-1.251	0.215
Model 6	Vividness	0.039 (1.426)	2.035	0.116	**(Constant)**	3.793 (0.772)		5.251	< 0.001 **
					TQ-R	0.587 (0.243)	0.271	2.417	0.018 *
					Age	0.010 (0.020)	0.056	0.501	0.618
					Ethnicity	-0.042 (0.144)	-0.033	-0.291	0.772
Model 7	Specificity	-0.006 (0.393)	0.848	0.472	**(Constant)**	0.634 (0.205)		3.092	0003 *
					POBS	-0.023 (0.029)	-0.088	-0.770	0.444
					Age	-0.004 (0.005)	-0.082	-0.709	0.480
					Ethnicity	-0.050 (0.040)	-0.146	-1.271	0.208
Model 8	Vividness	0.205 (1.297)	7.611	< 0.001 **	**(Constant)**	3.026 (0.677)		4.468	< 0.001 **
					POBS	0.461 (0.097)	0.483	4.745	< 0.001 **
					Age	0.003 (0.018)	0.018	0.177	0.860
					Ethnicity	-0.040 (0.131)	-0.031	-0.305	0.761

*Notes.*^a^experimenter rated, ^b^participant rated, *significant at *p* < 0.05, **significant at *p* < 0. 001. Specificity refers to the proportion of specific memories/future events. Abbreviations: POBS = The Perception of Betrayal Sensitivity; SE = standard error; TQ-R = Teasing Questionnaire- Revised; *ß* = standardised beta

## Discussion

To our knowledge, this is the first study investigating the specificity and vividness of disgust-related AMs and future events in both AN and binge-type EDs (BN or BED) compared to HC. Our first hypothesis, which predicted that participants with EDs would exhibit more difficulties in recalling specific autobiographical memories and constructing specific future events compared to HCs was not supported, as the proportion of specific AMs and EFTs was comparable between groups. Also, the proportion of specific AMs and future events did not vary depending on the valence of the cue (negative/moral disgust-relevant, or neutral). Future events primed by neutral cues were rated as more vivid by the control group compared to those induced by negative cues. Our second hypothesis predicted that the specificity of EFTs would be predicted by AM specificity, which was confirmed in only AN and HC groups. Exploratory analyses investigated the association between POBS and TQ-R scores, and the specificity and vividness of AMs and EFTs primed by negative/moral disgust-relevant cues. More self-reported experiences of childhood teasing, and greater betrayal sensitivity scores were associated with more vivid moral-disgust-related AMs and EFTs in people with EDs but not HCs.

Importantly, slight differences were observed in the findings when analyses were adjusted for DASS-Depression scores, suggesting that depressive symptoms may play a role in the relationship between ED psychopathology and characteristics of AMs and EFTSs. However, it is also possible that by adjusting for DASS-Depression scores, we may be inadvertently controlling for the effects of eating disorder psychopathology, as these variables were found to be highly correlated (*r* = 0.42) in the combined ED sample of the present study.

Previous research has employed a range of negative cue words, both general and ED specific. In the present study, we focused on negative cues related to disgust, specifically moral disgust - disgust directed at behaviours, individuals, or ideas that violate one’s moral, ethical, or social values. The manipulation check in the present study confirmed that all groups reported greater feelings of disgust towards AMs, and EFTs primed by negative/moral disgust-relevant cues than those primed by neutral cues. This evidence suggests that cue words (mistrust, exclusion, bullying, disloyalty, shame, and let-down) related to behaviours, individuals, or ideas that violate one’s moral, ethical, or social values can be employed in both AMT and EFT-T tasks to elicit disgust feelings. Furthermore, despite of comparable disgust sensitivity scores across all groups, we observed significant group differences in ratings of disgust in the AMT/EFT-T tasks between the ED groups and HC. In comparison with controls, participants with BN/BED reported greater disgust towards both AMs and EFTs, while participants with AN reported greater disgust towards only EFTs. Consistent with previous studies [26, 31, 52], our study also supported that disgust can be induced by internal sources such as autobiographical memories and episodic future thinking. Moreover, the disgust response to memories/future events primed by disgust-related words is greater in people with EDs compared to HCs [23, 29, 53, 54].

In contrast with the wider literature, we did not find evidence of overgeneral memory in people with EDs compared with controls. Previous research observed reductions in memory specificity in response to emotional cue words, in people with AN [6, 9, 13, 16]. A study by Keeler et al. [11] found that participants with AN recalled fewer specific AMs compared to controls, regardless of cue valence. However, our findings did not indicate such an overgeneral memory effect (OGM) in either the AN or the BN/BED groups compared to controls. This conflicting result may be due to differences in sociodemographic and clinical variables. For instance, in the present study, participants with AN were included if their BMI was ≥ 18.5 kg/m^2^ (*n* = 13; 30%), therefore a proportion of the AN sample may be better characterised as atypical AN. Participants in the present study also had a slightly higher average BMI (16.98 kg/m^2^) than in the study by Keeler et al. (16.02 kg/m^2^; mean BMI difference = 0.96 kg/m^2^) and in other studies (e.g., 14.7 kg/m^2^ [10], 15.5 kg/m^2^ [6]). In a meta-analysis of cognitive function in AN, BMI has been found to moderate cognitive performance with higher BMI resulting in smaller differences compared to controls, especially for the cognitive domain of memory [55]. The findings may also have been related to variations in the ethnicity between groups. More specifically, the proportion of Asian people (36.1%) in the HC group was significantly higher compared to AN (4.7%) and BN/BED (14.3%) groups. Previous evidence revealed that Asian people (i.e., Chinese, Chinese American, Taiwanese, or Japanese) tend to recall fewer specific AMs than Westerners [56, 57, 58, 59, 60]. However, all main analyses controlled for ethnicity and the findings were also robust in sensitivity analyses that excluded Asian participants from the sample. Finally, the findings may be explained by the absence of a positive cue word list in the present study. Some previous studies using positive, neutral and negative cue words have indicated nominally greater overgeneralisations for positive memories compared to negative or neutral memories [6, 11]. Therefore, this methodological difference may have contributed to our observed findings.

The findings in the present study also indicated that there was no significant difference in the ability to produce specific EFTs between groups, which is unsurprising given that AM specificity predicted EFT specificity in AN and HC groups, in keeping with the second hypothesis based on the Constructive Episodic Simulation hypothesis [32], and AM specificity was unaffected. Interestingly, we also found that HC group reported more vivid EFTs in response neutral cues compared to negative ones. This evidence may support the phenomenon known as the positivity bias, which refers to the idea that people are more likely to recall positive experiences and avoid negative ones [61]. As the HC group were not expected to have compromised AM or EFT, it makes sense that this group would demonstrate a positivity bias in future simulation. Imagining the future plays an important role in psychological well-being ​ [62]. In the control group, generating less vivid future events to negative cues may be considered vital in maintaining positive views of themselves and their personal future, constituting a self-protective mechanism. In contrast, this difference in vividness ratings between negative and neutral cues seen in HCs was not seen in EDs. It is possible that the presence of comorbid low mood in both ED groups, as seen in Table 2, may have negatively influenced future simulation [63]. It should also be highlighted that we did not ask participants to report another characteristics of EFTs, such as imageability; the capability of a cue to evoke mental images. Highly imageable cue words can evoke rich sensory experiences and emotional responses, making it easier for individuals to construct vivid and detailed mental representations of the future [64, 65]. It is possible that the imageability of the cue words differed between the neutral (book, table, chair, pen, window, and room) and the moral disgust-relevant (shame, exclusion, disloyalty, mistrust, bullying, let down) lists, which could have affected the ratings of vividness.

Additionally, our exploratory regression analyses indicated a positive association between childhood experiences of teasing and betrayal sensitivity and the self-reported vividness of AMs and EFTs in response to cues representing negative life events violating personal, social, or moral values. This association was found only in the ED group. Both teasing and bullying have been found to have similar effect on increasing the risk for disordered eating behaviours [66]. Lie and colleagues [67] conducted separate meta-analyses for generic bullying, appearance-unrelated teasing, and appearance-related teasing. Their findings revealed that, compared to HCs, individuals with EDs were two-to-three times more likely to have been teased about their appearance and bullied prior to onset of their EDs. The authors posited that the relationship between such stressful events and EDs could be explained through several interrelated mechanisms, including heightened emotional distress (e.g., shame, anxiety, isolation), increased preoccupation with appearance, social comparison and body dissatisfaction, all of which exacerbate maladaptive coping strategies such as dietary restraint and binge-eating. However, to the best of our knowledge, this is the first study to examine the role of betrayal sensitivity in the ED population. Betrayal sensitivity has been reported to influence behaviour alongside expectations of trustworthiness [68], occurring transdiagnostically [69]. It is possible that these victimisation experiences are more salient in one’s mind and therefore more vivid and may be more salient to people with EDs with a higher impact on imagined negative future events [70] given the evidence that they frequently report a variety of fears of experiencing weight gain, loss of control, and judgment by others [71, 72]. The directionality of the association is unclear; for example, it is also likely that the increased vividness of memories relating to such negative life events may contribute to greater betrayal sensitivity. Furthermore, these results should be considered with caution given their exploratory and preliminary nature.

### Strengths and limitations of the present study

Study strengths include that the sample included individuals with binge-type EDs. Another strength is that we used a PPI group to generate salient negative cues to address moral disgust. There are however several limitations to this study. First, the findings should be regarded as preliminary due to a relatively small sample size and cross-sectional nature of the study. Ideally, it would have been even better to have a separate BN and BED group, but this was not possible because of recruitment challenges. Second, to verify the clinical significance of ED symptomatology, self-report questionnaires were used rather than structured interviews, and BMI measures were based on self-reported weight and height and therefore could not be verified by researchers. Third, the remote administration of the tasks might be considered another limitation since the environment in which participants completed the tasks might be more variable. Given the low number of omissions per group (*n* = 0 for HC, *n* = 3 for AN, *n* = 2 for BN/BED; see Table S3), we might imply that participants paid enough attention and/or engaged with the online tasks. However, due to online nature of the study, it is difficult to exactly acquire the reasons behind omissions and control for potential distractions for each participant. Also, in the present study, participants were given two minutes to write down a specific personal memory and future event for each of the cue words, which could be considered a longer time period compared to previous research varying between 30 and 60 s. It is arguable that a longer duration per cue could prevent the detection of significant group differences in specificity, although other studies have found differences in AM or EFT specificity using a written or computerised version of AMT with both a 2-minute response time [11, 70] or no time limit at all [59]. Fourth, the absence of an assessment of alexithymia is another limitation in the present study. Previous studies [6, 13] have reported an overgeneral memory effect in people with AN irrespective of the presence of alexithymia. In contrast, Apgáua and Jaeger [73] reviewed thirteen studies investigating memory for emotional information in individuals with different levels of alexithymia and found evidence that individuals with high alexithymia exhibit a reduced ability to encode and recall emotional information. However, the authors suggested interpreting findings of this systematic review with caution since the number of studies (*n* = 2 studies where AMT was utilized) was relatively small. Incorporating measures of alexithymia would allow us to better understand and control its potential influence on the on AM recall and EFT construction in people with and without EDs. Lastly, ethnic/racial, and age-related differences between groups were observed, although this was controlled for in all analyses and in sensitivity analyses. While only DASS-Depression scores were controlled in this study due to the high comorbidity between major depression and EDs, it is important to note that individuals with EDs often have additional comorbidities, such as autism spectrum disorder (ASD), which is associated with differences in socio-cognition and executive functioning. In the present study, six participants with EDs (≈ 7%) reported a comorbid diagnosis of ASD. As reported in a recent comprehensive thematic review [74], individuals with ASD tend to recall significantly fewer and less detailed AMs particularly in response to emotional cues and take longer to do so compared to neurotypical control groups. It is possible that general difficulties in the cognitive processing of emotions, such as identifying and naming emotions, which are often observed in ASD [75], could impact memory characteristics (i.e., detail and emotional intensity). In this regard, the comorbidity of ASD in our ED cohort might have also influenced our findings. However, as the diagnosis was self-reported and the prevalence was relatively low, it is outside of the scope of this study to interrogate this further. Further replication studies should examine the possible effects of the comorbidity of ASD with EDs on AM and EFT, using a more detailed diagnostic assessment.

### Future research

To make our findings more generalisable, research within more diverse communities and from various continents is needed [76]. More studies following a more standardised methodology is required to better delineate the effects of illness parameters (i.e., BMI, illness duration, depression level, comorbidity, social adjustment) on AM recall and EFT construction. Recruiting samples from one particular treatment modalities, for example, inpatient units, would be of benefit as would matching groups according to socio-demographic characteristics (e.g., ethnicity, age) as a recruitment strategy to minimise variation in such characteristics between groups. It would be preferable to have a larger comparison group in order to match the diverse characteristics of the groups. It may also be more important for researchers who will code the specificity of memories/future events to be blind to the purpose of the study rather than the participants’ diagnostic status. Since the AMT relies heavily on participants’ ability to access and articulate internal emotional states, future research could also consider incorporating measures of alexithymia, such as the Toronto Alexithymia Scale (TAS-20) [77]. Future research could consider incorporating positive cue words alongside neutral and moral disgust-relevant cue words and assessing the imageability of each cue word prior to the experiment. This would allow for the matching of cues with similar levels of imageability.

AMs, future events, and their interpretation might change during psychological therapy. Thus, the influence of psychotherapy on disgust elicited by memories/future events and the perception of trauma and betrayal might be a further area for research. Qualitative research exploring memories and future events produced in response to moral disgust-relevant cues would be invaluable for clinical practice to understand the content and nature of memories/future events rather than only a single metric of specificity. Finally, longitudinal cohort studies starting from school age are needed to establish causal relationships between experiences of teasing, betrayal (actual and/or perceived), relevant memories/future events and the development of ED-specific and general psychopathology.

## Conclusions

This study did not find evidence of overgeneralisation for memories or future events in individuals with EDs compared to controls, in response to disgust-related and neutral cues. Further research is needed to clarify the role of clinical features such as illness severity on the specificity of AMs and future events. Interestingly, only the control group reported more vivid future events in response to neutral cues, potentially reflecting a positivity bias in future simulation. Alternatively, it is possible that low mood, which is observed in people with EDs, may negatively impact future event simulation. Conversely, childhood teasing, and betrayal sensitivity were associated with more vivid disgust-inducing memories and future envisioning in the ED sample, highlighting the potential influence of victimisation experiences on experiences of past and future events.

## Electronic supplementary material

Below is the link to the electronic supplementary material.

Supplementary Material 1

## Data Availability

No datasets were generated or analysed during the current study.

## References

[1] Mitchell JE, Wonderlich SA. Feeding and eating disorders. The American Psychiatric Publishing textbook of psychiatry. 6th ed. Arlington, VA, US: American Psychiatric Publishing, Inc.; 2014. pp. 557–86. 10.1176/appi.books.9781585625031.rh17.

[2] Fossati P. Imaging autobiographical memory. Dialogues Clin Neurosci. 2013;15(4). 10.31887/dcns.2013.15.4/pfossati. 487– 90. 10.31887/DCNS.2013.15.4/pfossati PMC3898686 24459415

[3] Nawa NE, Ando H. Effective connectivity within the ventromedial prefrontal cortex-hippocampus-amygdala network during the elaboration of emotional autobiographical memories. NeuroImage. 2019;189:316–28. 10.1016/j.neuroimage.2019.01.042. 30665009 10.1016/j.neuroimage.2019.01.042

[4] Vanderveren E, Bijttebier P, Hermans D. The importance of memory specificity and memory coherence for the self: linking two characteristics of autobiographical memory. Front Psychol. 2017;8:2250. 10.3389/fpsyg.2017.02250. 29312089 10.3389/fpsyg.2017.02250 PMC5744072

[5] Barry TJ, Hallford DJ, Takano K. Autobiographical memory impairments as a Transdiagnostic feature of Mental illness: a Meta-Analytic Review of investigations into Autobiographical Memory specificity and overgenerality among people with Psychiatric diagnoses. Psychol Bull. 2021;147(10):1054. 10.31234/osf.io/ab5cu. 34968086 10.1037/bul0000345

[6] Bomba M, Marfone M, Brivio E, Oggiano S, Broggi F, Neri F, et al. Autobiographical memory in adolescent girls with anorexia nervosa. Eur Eat Disord Rev. 2014;22(6):479–86. 10.1002/erv.2321. 25267565 10.1002/erv.2321

[7] Brockmeyer T, Grosse Holtforth M, Bents H, Herzog W, Friederich HC. Lower body weight is associated with less negative emotions in sad autobiographical memories of patients with anorexia nervosa. Psychiatry Res. 2013;210(2):548–52. 10.1016/j.psychres.2013.06.024. 23850436 10.1016/j.psychres.2013.06.024

[8] Castellon P, Sudres JL, Voltzenlogel V. Self-defining memories in female patients with anorexia nervosa. Eur Eat Disord Rev. 2020;28(5):513–24. 10.1002/erv.2739. 32363663 10.1002/erv.2739

[9] Dalgleish T, Yiend J, Tchanturia K, Serpell L, Hems S, De Silva P, et al. Self-reported parental abuse relates to autobiographical memory style in patients with eating disorders. Emotion. 2003;3(3):211–22. 10.1037/1528-3542.3.3.211. 14498792 10.1037/1528-3542.3.3.211

[10] Huber J, Salatsch C, Ingenerf K, Schmid C, Maatouk I, Weisbrod M, et al. Characteristics of disorder-related autobiographical memory in Acute Anorexia Nervosa patients. Eur Eat Disord Rev. 2015;23(5):379–89. 10.1002/erv.2379. 26095135 10.1002/erv.2379

[11] Keeler JL, Peters-Gill G, Treasure J, Himmerich H, Tchanturia K, Cardi V. Difficulties in retrieving specific details of autobiographical memories and imagining positive future events in individuals with acute but not remitted anorexia nervosa. J Eat Disord. 2022;10(1):172. 10.1186/s40337-022-00684-w. 36401319 10.1186/s40337-022-00684-w PMC9675114

[12] Laberg S, Andersson G. Autobiographical memories in patients treated for bulimia nervosa. Eur Eat Disord Rev. 2004;12(1):34–41. 10.1016/j.jpsychores.2006.02.008.

[13] Nandrino JL, Doba K, Lesne A, Christophe V, Pezard L. Autobiographical memory deficit in anorexia nervosa: emotion regulation and effect of duration of illness. J Psychosom Res. 2006;61(4):537–43. 10.1016/j.jpsychores.2006.02.008. 17011363 10.1016/j.jpsychores.2006.02.008

[14] Rasmussen AS, Jørgensen CR, O’Connor M, Bennedsen BE, Godt KD, Bøye R, et al. The structure of past and future events in borderline personality disorder, eating disorder, and obsessive-compulsive disorder. Psychol Conscious. 2017;4(2):190. 10.1037/cns0000109.

[15] Terhoeven V, Faschingbauer S, Huber J, Herzog W, Friederich HC, Simon JJ, et al. Verbal memory following weight gain in adult patients with anorexia nervosa: a longitudinal study. Eur Eat Disord Rev. 2023;31(2):271–84. 10.1002/erv.2956. 36397677 10.1002/erv.2956

[16] Kovács T, Szabó P, Pászthy B. Reduced specificity of autobiographical memory in anorexia nervosa. J Behav Cogn Ther. 2011;11(1):57–66. https://www.proquest.com/scholarly-journals/reduced-specificity-autobiographical-memory/docview/868867760/se-2.

[17] Ridout N, Matharu M, Sanders E, Wallis DJ. The influence of eating psychopathology on autobiographical memory specificity and social problem-solving. Psychiatry Res. 2015;228(3):295–303. 10.1016/j.psychres.2015.06.030. 26144580 10.1016/j.psychres.2015.06.030

[18] Mang L, Ridout N, Dritschel B. The influence of mood and attitudes towards eating on cognitive and autobiographical memory flexibility in female university students. Psychiatry Res. 2018;269:444–9. 10.1016/j.psychres.2018.08.055. 30195233 10.1016/j.psychres.2018.08.055

[19] Au Yeung C, Dalgleish T, Golden AM, Schartau P. Reduced specificity of autobiographical memories following a negative mood induction. Behav Res Ther. 2006;44(10):1481–90. 10.1016/j.brat.2005.10.011. 16356472 10.1016/j.brat.2005.10.011

[20] Forester G, Johnson JS, Reilly EE, Lloyd EC, Johnson E, Schaefer LM. Back to the future: progressing memory research in eating disorders. Int J Eat Disord. 2023;56(11):2032–48. 10.1002/eat.24045. 37594119 10.1002/eat.24045 PMC10843822

[21] Williams JMG, Barnhofer T, Crane C, Hermans D, Raes F, Watkins E, et al. Autobiographical memory specificity and emotional disorder. Psychol Bull. 2007;133(1):122. 10.1037/0033-2909.133.1.122. 17201573 10.1037/0033-2909.133.1.122 PMC2834574

[22] Scharner S, Stengel A. Alterations of brain structure and functions in anorexia nervosa. Clin Nutr Exp. 2019;28:22–32. 10.1016/j.yclnex.2019.02.001.

[23] Bektas S, Keeler JL, Anderson LM, Mutwalli H, Himmerich H, Treasure J. Disgust and self-disgust in eating disorders: a systematic review and Meta-analysis. Nutrients. 2022;14(9):1728. 10.3390/nu14091728. 35565699 10.3390/nu14091728 PMC9102838

[24] Bektas S, Natali L, Rowlands K, Valmaggia L, Di Pietro J, Mutwalli H, et al. Exploring correlations of Food-Specific Disgust with Eating Disorder psychopathology and Food Interaction: a preliminary Study using virtual reality. Nutrients. 2023;15(20):4443. 10.3390/nu15204443. 37892518 10.3390/nu15204443 PMC10609698

[25] Bektas S, Himmerich H, Treasure J. Genetic and Environmental Aspects of Eating Disorders. In: Eating Disorders: An Internal Comprehensive View. 2023;1–13. 10.1007/978-3-030-97416-9_34-1

[26] Ozawa S, Nakatani H, Miyauchi CM, Hiraki K, Okanoya K. Synergistic effects of disgust and anger on amygdala activation while recalling memories of interpersonal stress: an fMRI study. Int J Psychophysiol. 2022;182:39–46. 10.1016/j.ijpsycho.2022.09.008. 36167180 10.1016/j.ijpsycho.2022.09.008

[27] Udo T, Grilo CM. Prevalence and correlates of DSM-5–Defined eating disorders in a nationally Representative Sample of United States adults. Biol Psychiatry. 2018;84(5):345–54. 10.1016/j.biopsych.2018.03.014. 29859631 10.1016/j.biopsych.2018.03.014 PMC6097933

[28] Grogan K, MacGarry D, Bramham J, Scriven M, Maher C, Fitzgerald A. Family-related non-abuse adverse life experiences occurring for adults diagnosed with eating disorders: a systematic review. J Eat Disord. 2020;8(1):36. 10.1186/s40337-020-00311-6. 32704372 10.1186/s40337-020-00311-6 PMC7374817

[29] von Spreckelsen P, Wessel I, Glashouwer KA, de Jong PJ. Averting repulsion? Body-Directed Self-Disgust and Autobiographical Memory Retrieval. J Exp Psychopathol. 2022;13(1). 10.31234/osf.io/qhc35.

[30] von Spreckelsen P, Wessel I, Glashouwer KA, de Jong PJ. Negative body image and avoidant retrieval of body-related autobiographical memories. Memory. 2023;31(2):192–204. 10.1080/09658211.2022.2135734. 36269098 10.1080/09658211.2022.2135734

[31] von Spreckelsen P, Wessel I, Glashouwer KA, de Jong PJ. Escaping from revulsion - disgust and escape in response to body-relevant autobiographical memories. Memory. 2022;30(2):104–16. 10.1080/09658211.2021.1993923. 34762021 10.1080/09658211.2021.1993923

[32] Schacter DL, Addis DR. The cognitive neuroscience of constructive memory: remembering the past and imagining the future. Philosophical Trans Royal Soc B: Biol Sci. 2007;362(1481):773–86. 10.1098/rstb.2007.2087. 10.1098/rstb.2007.2087 PMC2429996 17395575

[33] Bohon C, Stice E. Eating Disorder Diagnostic Scale. In: Encyclopedia of Feeding and Eating Disorders. 2017;254-7. 10.1007/978-981-287-104-6_109

[34] Stice E, Telch CF, Rizvi SL. Development and validation of the eating disorder diagnostic scale: a brief self-report measure of anorexia, bulimia, and binge-eating disorder. Psychol Assess. 2000;12(2):123–31. 10.1037//1040-3590.12.2.123. 10.1037//1040-3590.12.2.123 10887758

[35] Ono M, Devilly GJ, Shum DHK. A Meta-Analytic Review of Overgeneral Memory: the role of Trauma History, Mood, and the Presence of Posttraumatic stress disorder. Psychol Trauma. 2016;8(2):157–64. 10.1037/tra0000027. 25961867 10.1037/tra0000027

[36] Calugi S, Pace CS, Muzi S, Fasoli D, Travagnin F, Dalle Grave R. Psychometric proprieties of the Italian version of the questionnaire on eating and weight patterns (QEWP-5) and its accuracy in screening for binge-eating disorder in patients seeking treatment for obesity. Eat Weight Disord. 2020;25(6):1739–45. 10.1007/s40519-019-00818-1. 31784945 10.1007/s40519-019-00818-1

[37] Fairburn CG, Beglin S. Eating disorder examination questionnaire. Fairburn C.G., editor. New York, NY: Guilford Press. 2008;309–313. 10.1037/t03974-000

[38] Gormally J, Black S, Daston S, Rardin D. The assessment of binge eating severity among obese persons. Addict Behav. 1982;7(1):47–55. 10.1016/0306-4603(82)90024-7. 7080884 10.1016/0306-4603(82)90024-7

[39] Lovibond SH, Lovibond PF. Manual for the Depression anxiety stress scales. Psychol Foundation Australia. 1995;33(3):335–43. 10.1037/t01004-000. 10.1016/0005-7967(94)00075-u 7726811

[40] Olatunji B, Wadkins M, Mckay D, Bjorklund F, Jong P, Haidt J, et al. Confirming the three-factor structure of the Disgust scale–revised in eight countries. J Cross Cult Psychol. 2009;40(2):234–55. 10.1177/0022022108328918.

[41] Powell PA, Overton PG, Simpson J. The revolting self: perspectives on the psychological, social, and clinical implications of self-directed disgust. Powell PA, Overton PG, Simpson J, editors. Routledge. 2018. 10.4324/9780429483042

[42] Storch E, Roth D, Coles M, Heimberg R, Bravata E, Moser J. The measurement and impact of childhood teasing in a sample of young adults. J Anxiety Disord. 2004;18(5):681–94. 10.1016/j.janxdis.2003.09.003. 15275946 10.1016/j.janxdis.2003.09.003

[43] Pagdin R, Salkovskis PM, Nathwani F, Wilkinson-Tough M, Warnock-Parkes E. I was treated like dirt: evaluating links between betrayal and mental contamination in clinical samples. Behav Cogn Psychother. 2021;49(1):21–34. 10.1017/s1352465820000387. 32854797 10.1017/S1352465820000387

[44] Mancini A, Granziol U, Migliorati D, Gragnani A, Femia G, Cosentino T, et al. Moral Orientation Guilt Scale (MOGS): development and validation of a novel guilt measurement. Pers Individ Dif. 2022;189:111495. 10.1016/j.paid.2021.111495.

[45] Barry TJ, Takano K, Boddez Y, Raes F. Lower sleep duration is Associated with reduced autobiographical memory specificity. Behav Sleep Med. 2019;17(5):586–94. 10.1080/15402002.2018.1435542. 29424553 10.1080/15402002.2018.1435542

[46] Johns MW. A new method for measuring daytime sleepiness: the Epworth sleepiness scale. Sleep. 1991;14(6):540–5. 10.1093/sleep/14.6.540. 1798888 10.1093/sleep/14.6.540

[47] Williams JMG, Broadbent K. Autobiographical memory in suicide attempters. J Abnorm Psychol. 1986;95(2):144–9. 10.1037//0021-843x.95.2.144. 10.1037//0021-843x.95.2.144 3711438

[48] Hallford DJ, Takano K, Raes F, Austin DW. Psychometric evaluation of an episodic future thinking variant of the autobiographical memory test - episodic future thinking-test (EFT-T). Eur J Psychol Assess. 2020;36(4). 10.31234/osf.io/5uyba.

[49] Powell P, Consedine N. The Handbook of Disgust Research Modern Perspectives and Applications. 2021. 10.1007/978-3-030-84486-8

[50] Anwyl-Irvine AL, Massonnié J, Flitton A, Kirkham N, Evershed JK. Gorilla in our midst: an online behavioral experiment builder. Behav Res Methods. 2020;52(1):388–407. 10.3758/s13428-019-01237-x. 31016684 10.3758/s13428-019-01237-x PMC7005094

[51] IBM Corp. Released. IBM SPSS Statistics version 27.0. 2020;15–24. 10.4324/9781003117452-4

[52] Bø S, Norman E, Wolff K. Discrete emotions caused by episodic future thinking: a systematic review with narrative synthesis. Collabra Psychol. 2022;8(1):35232. 10.1525/collabra.35232.

[53] von Spreckelsen P, De Jong PJ. Disgust-induced avoidant processing of autobiographical memories as a transdiagnostic mechanism in the persistence of psychopathology. Bull Menn Clin. 2023;87:31–52. 10.1521/bumc.2023.87.suppa.31. 10.1521/bumc.2023.87.suppA.31 37871194

[54] von Spreckelsen Ineke Wessel KAG, de Jong PJ. Escaping from revulsion - disgust and escape in response to body-relevant autobiographical memories. Memory. 2022;30(2):104–16. 10.1080/09658211.2021.1993923. 34762021 10.1080/09658211.2021.1993923

[55] Stedal K, Broomfield C, Hay P, Touyz S, Scherer R. Neuropsychological functioning in adult anorexia nervosa: a meta-analysis. Neurosci Biobehav Rev. 2021;130:214–26. 10.1016/j.neubiorev.2021.08.021. 34453951 10.1016/j.neubiorev.2021.08.021

[56] Wang Q. Once upon a time: explaining Cultural differences in episodic specificity. Soc Personal Psychol Compass. 2009;3(4):413–32. 10.1111/j.1751-9004.2009.00182.x.

[57] Millar PR, Serbun SJ, Vadalia A, Gutchess AH. Cross-cultural differences in memory specificity. Cult Brain. 2013;1(2–4):138–5. 10.1007/s40167-013-0011-3.

[58] Dritschel B, Kao CM, Astell A, Neufeind J, Lai TJ. How are depression and autobiographical memory retrieval related to culture? J Abnorm Psychol. 2011;120(4):969–74. 10.1037/a0025293. 21942336 10.1037/a0025293

[59] Takano K, Mori M, Nishiguchi Y, Moriya J, Raes F. Psychometric properties of the written version of the autobiographical memory test in a Japanese community sample. Psychiatry Res. 2017;248:56–63. 10.1016/j.psychres.2016.12.019. 28013087 10.1016/j.psychres.2016.12.019

[60] Hallford DJ, Barry TJ, Belmans E, Raes F, Dax S, Nishiguchi Y, et al. Specificity and detail in autobiographical memory retrieval: a multi-site (re)investigation. Memory. 2021;29(1):1–10. 10.1080/09658211.2020.1838548. 33135956 10.1080/09658211.2020.1838548

[61] Marsh L, Edginton T, Conway MA, Loveday C. Positivity bias in past and future episodic thinking: relationship with anxiety, depression, and retrieval-induced forgetting. Q J Exp Psychol. 2019;72(3):508–22. 10.1177/1747021818758620. 10.1177/1747021818758620 29364056

[62] Schubert T, Eloo R, Scharfen J, Morina N. How imagining personal future scenarios influences affect: systematic review and meta-analysis. Clin Psychol Rev. 2020;75:75:101811. 10.1016/j.cpr.2019.101811. 31884148 10.1016/j.cpr.2019.101811

[63] Hallford DJ, Austin DW, Takano K, Raes F. Psychopathology and episodic future thinking: a systematic review and meta-analysis of specificity and episodic detail. Behav Res Ther. 2018;102:42–51. 10.1016/j.brat.2018.01.003. 29328948 10.1016/j.brat.2018.01.003

[64] Rasmussen KW, Berntsen D. I can see clearly now: the effect of cue imageability on mental time travel. Mem Cognit. 2014;42(7):1063–75. 10.3758/s13421-014-0414-1. 24874508 10.3758/s13421-014-0414-1

[65] Ballot C, Robert C, Mathey S. Word imageability influences the emotionality effect in episodic memory. Cogn Process. 2022;23(4):655–60. 10.1007/s10339-022-01102-4. 35857171 10.1007/s10339-022-01102-4 PMC9553820

[66] Day S, Bussey K, Trompeter N, Mitchison D. The impact of teasing and bullying victimization on disordered eating and body image disturbance among adolescents: a systematic review. Trauma Violence Abuse. 2022;23(3):985–1006. 10.1177/1524838020985534. 33461439 10.1177/1524838020985534

[67] Lie S, Bulik CM, Andreassen OA, Rø Ø, Bang L. Stressful life events among individuals with a history of eating disorders: a case-control comparison. BMC Psychiatry. 2021;21(1):1–2. 10.1186/s12888-021-03499-2. 34645394 10.1186/s12888-021-03499-2 PMC8513319

[68] Thielmann I, Hilbig BE, Trust. An integrative review from a person-situation perspective. Rev Gen Psychol. 2015;19(3):249–77. 10.1037/gpr0000046.

[69] Howkins S, Millar JFA, Salkovskis PM. Sensitivity to being betrayed and betraying others in obsessive compulsive disorder and depression. Br J Clin Psychol. 2022;61(1):58–75. 10.1111/bjc.12319. 34269428 10.1111/bjc.12319

[70] Bunnell SL, Legerski JP, Herting NR. The autobiographical memory test: differences in memory specificity across three recall elicitation methods. Curr Psychol. 2020;39(6):2298–305. 10.1007/s12144-018-9930-7.

[71] Brown ML, Levinson CA. Core eating disorder fears: prevalence and differences in eating disorder fears across eating disorder diagnoses. Int J Eat Disord. 2022;55(7):956–65. 10.1186/s40337-023-00745-8. 35567750 10.1002/eat.23728

[72] Melles H, Jansen A. Transdiagnostic fears and avoidance behaviors in self-reported eating disorders. J Eat Disord. 2023;11(1):19. 10.1186/s40337-023-00745-8. 36782316 10.1186/s40337-023-00745-8 PMC9926724

[73] Apgáua LT, Jaeger A. Memory for emotional information and alexithymia: a systematic review. Dement Neuropsychol. 2019;13(1):22–30. 10.1590/1980-57642018dn13-010003. 31073377 10.1590/1980-57642018dn13-010003 PMC6497026

[74] Griffin JW, Bauer R, Gavett BE. The Episodic Memory Profile in Autism Spectrum disorder: a bayesian Meta-analysis. Neuropsychol Rev. 2022;32(2):316–51. 10.1007/s11065-021-09493-5. 33954915 10.1007/s11065-021-09493-5

[75] Hill E, Berthoz S, Frith U. Brief report: cognitive processing of own emotions in individuals with autistic spectrum disorder and in their relatives. J Autism Dev Disord. 2004;34(2):229–35. 10.1023/b:jadd.0000022613.41399.14. 15162941 10.1023/b:jadd.0000022613.41399.14

[76] Himmerich H, Keeler J, Davies H, Tessema SA, Treasure J. The evolving profile of eating disorders and their treatment in a changing and globalised world. Lancet. 2024;403(10445):2761–5. 10.1016/s0140-6736(24)00874-2. 38705161 10.1016/S0140-6736(24)00874-2

[77] Parker JDA, Taylor GJ, Bagby RM. The 20-Item Toronto Alexithymia Scale: III. Reliability and factorial validity in a community population. J Psychosom Res. 2003;55(3). 10.1016/s0022-3999(02)00601-3. 10.1016/s0022-3999(02)00578-0 12932802

