# Supplementary material for: An investigation of the specificity and vividness of autobiographical memories and future events produced in response to disgust-related cues among individuals with eating disorders
Source: J Eat Disord. 2025 Feb 24;13:39. doi: 10.1186/s40337-025-01214-0 (PMC11853898; doi:10.1186/s40337-025-01214-0)
Supplement: Supplementary file 1 — Supplementary Material 1 [file 40337_2025_1214_MOESM1_ESM.docx]

**Supplementary Materials**

**Table S1.** Autobiographical memory test and episodic future thinking task instructions

| **Autobiographical Memory Task** |
| --- |
| You will now be presented with a series of 6 words, one at a time. Your task will be to describe a specific event that happened to you in the past (from a week ago or longer). The event does not have to be directly related to this word but may be just inspired by it. Here are some instructions for what type of past event to describe:   - It should be something you were personally involved in - It should be something that occurred in a particular place and within the space of a day at most. - Please try and state as many details about this past event as you can (e.g. where it happened, who was there, what happened, how you felt, what you thought etc.). - Please describe a different event for each word.   You will be given 2 minutes to respond. You will then be asked to rate this memory on a series of scales. Please click continue for some examples of responses to this task |
| If the word was “UPSET”, then an example of a response would be:  A few weeks ago, my partner and I had a big fight. We were at home, and we had a disagreement about the housework. I felt very stressed and sad and went for a walk to take my mind off it. I remember thinking that the relationship was not great at that time.  If the word was “HOUSE”, then an example of a response would be:  Last week I went to view a house, as I am thinking about moving. It was in the perfect location for work and looked really nice inside, but it is slightly out of my budget. I felt apprehensive about making any decisions on whether to put an offer in and thought I should wait and see what else comes up on the market. |
| Please press the button below to continue to the task. You will have 2 minutes per word to write your response. Remember to try and state as many details about this past event as you can (e.g. where it happened, who was there, what happened, how you felt, what you thought etc.). Please describe a different event for each word. |
| **Episodic Future Thinking Task** |
| You will now be presented with a series of 6 words, one at a time. Your task will be to describe a specific event that could happen in your future. The event does not have to be directly related to this word but may be just inspired by it. Here are some instructions for what type of future event to describe:   - It should be something you would be personally involved in - It should be something that occurs in a particular place and within the space of a day at most. - Please try and state as many details about this possible future event as you can (e.g. where it happens, who would be there, what would happen, how you might feel, what you might think etc.). - Please describe a different event for each word.   You will be given 2 minutes to respond. You will then be asked to rate this future event on a series of scales. Please click continue for some examples of responses to this task. |
| If the word was: “PLEASURE”, then an example of a response would be:  I have a friend's birthday party at their house next month. The house is big, but I would spend most of my time in their kitchen. A lot of other friends will be there, and there will be dancing and music. It will probably be a late night, and I'll eat many of the snacks.  If the word was “TABLE”, then an example of a response would be:  I can imagine one day having to stand up on my table at home to fix a light. I'll be a little worried about the height and that I might fall off. I'll change the light and then get down off the table. I'll feel proud that I managed to do it. |
| Please press the button below to continue to the task. You will have 2 minutes per word to write your response. Remember to try and state as many details about this future event as you can (e.g. where it happens, who would be there, what would happen, how you might feel, what you might think etc.). Please describe a different event for each word. |

**Table S2.** Negative cue words given to PPI group (n = 15) to rate how much disgusted and violated they feel.

| Negative Cue Words | Mean for disgust | Mean for violation |
| --- | --- | --- |
| Betrayal | 3.67 | 4.33 |
| Teasing | 2.53 | 2.20 |
| Mistrust | 3.93 | 4.00 |
| Shunned | 2.93 | 3.40 |
| Exclusion | 4.00 | 3.36 |
| Bullying | 3.67 | 3.87 |
| Mocking | 3.53 | 2.80 |
| Disloyalty | 4.07 | 4.27 |
| Shame | 4.20 | 3.60 |
| Let Down | 3.73 | 3.47 |

**Table S3.** Descriptive characteristics of autobiographical memories/future events per cue type and group.

|  | **HC (n = 36)** | **AN (n = 43)** | **BN/BED (n = 35)** |
| --- | --- | --- | --- |
| **AMT – neutral cue words n (%)** | | | |
| Specific | 58 (45%) | 89 (69%) | 72 (55.8%) |
| Extended | 20 (15.5%) | 14 (10.9%) | 18 (14%) |
| Categoric | 20 (15.5%) | 22 (17.1%) | 12 (9.3%) |
| Semantic | 2 (1.6%) | - | 1 (0.8%) |
| Off-task | 8 (6.2%) | 4 (3.1%) | 2 (1.6%) |
| Omission | - | - | - |
| **AMT – negative cue words n (%)** | | | |
| Specific | 57 (44.2%) | 77 (59.7%) | 66 (51.2%) |
| Extended | 9 (7%) | 11 (8.5%) | 18 (14%) |
| Categoric | 14 (10.9%) | 18 (14%) | 12 (9.3%) |
| Semantic | 13 (10.1%) | 13 (10.1%) | 2 (1.6%) |
| Off-task | 15 (11.6%) | 9 (7%) | 5 (3.9%) |
| Omission | - | 1 (0.8%) | 2 (1.6%) |
| **EFT-T neutral cue words n (%)** | | | |
| Specific | 65 (50.4%) | 76 (58.9%) | 52 (40.3%) |
| Extended | 29 (22.5%) | 33 (25.6%) | 35 (27.1%) |
| Categoric | 8 (6.2%) | 11 (8.5%) | 8 (6.2%) |
| Semantic | 1 (0.8%) | 3 (2.3%) | 1 (0.8%) |
| Off-task | 5 (3.9%) | 6 (4.7%) | 9 (7%) |
| Omission | - | - | - |
| **EFT-T negative cue words n (%)** | | | |
| Specific | 38 (29.5) | 49 (38%) | 52 (40.3%) |
| Extended | 16 (12.4) | 32 (24.8%) | 35 (27.1%) |
| Categoric | 12 (9.3%) | 16 (12.4%) | 8 (6.2%) |
| Semantic | 28 (21.7%) | 20 (15.5%) | 1 (0.8%) |
| Off-task | 14 (10.9%) | 10 (7.8%) | 9 (7%) |
| Omission | - | 2 (1.6%) | - |

*Notes.* AMT = Autobiographical Memory Task; AN = Anorexia Nervosa; BN/BED = Bulimia Nervosa and Binge Eating Disorders; EFT-T = Episodic Future Thinking Task; HC = Healthy Controls.

**Table S4.** Effect of cue valence, group, and cue valence x group in ANCOVA models for autobiographical memory test (AMT) and episodic future thinking task (EFT-T) outcomes after controlling for DASS-Depression scores.

| Outcome | Cue Valence | | Group | | Cue Valence x Group | |
| --- | --- | --- | --- | --- | --- | --- |
|  | **F-value (1,109)** | ***p* value (η2)** | **F-value (2,109)** | ***p* value (η2)** | **F-value (3,109)** | ***p* value (η2)** |
| *Autobiographical Memory Test* | | | | | | |
| Specificity ^a^ | 0.177 | 0.675 (0.002) | 1.069 | 0.347 (0.019) | 1.144 | 0.322 (0.021) |
| Vividness ^b^ | 2.395 | 0.125 (0.022) | 1.900 | 0.155 (0.034) | 0.096 | 0.908 (0.002) |
| Disgust ^b^ | 5.501 | 0.021 ** (0.048) | 1.142 | 0.323 (0.021) | 2.350 | 0.100 (0.042) |
| *Episodic Future Thinking Task* | | | | | | |
| Specificity ^a^ | 1.213 | 0.273 (0.011) | 0.228 | 0.796 (0.004) | 1.379 | 0.716 (0.006) |
| Vividness ^b^ | 9.980 | 0.002 ** (0.085) | 1.005 | 0.370 (0.018) | 0.917 | 0.403 (0.017) |
| Disgust ^b^ | 11.242 | 0.001 ** (0.094) | 0.422 | 0.657 (0.008) | 1.966 | 0.145 (0.035) |

*Notes.* * Significant at the *p* < 0.05 threshold, ** Significant at the *p* < 0.01 threshold. *** Significant at the *p* < 0.001 threshold. ^a^ Researcher rated ^b^ Participant rated. Specificity refers to the proportion of specific autobiographical memories/future events. All analyses were run with age, ethnicity and DASS-Depression scores entered as covariates.

**Table S5.** Effect of cue valence, group, and cue valence x group in ANCOVA models for autobiographical memory test (AMT) and episodic future thinking task (EFT-T) outcomes in the sample without Asian participants (HC n = 23; AN n = 41; BN/BED n = 30).

| Outcome | Cue Valence | | Group | | Cue Valence x Group | |
| --- | --- | --- | --- | --- | --- | --- |
|  | **F-value (1,88)** | ***p* value (η2)** | **F-value (2,88)** | ***p* value (η2)** | **F-value (3,88)** | ***p* value (η2)** |
| *Autobiographical Memory Test* | | | | | | |
| Specificity ^a^ | 0.284 | 0.595  (0.003) | 0.626 | 0.537  (0.014) | 2.436 | 0.093  (0.052) |
| Vividness ^b^ | 0.092 | 0.763  (0.001) | 1.678 | 0.193  (0.037) | 0.326 | 0.723  (0.007) |
| Disgust ^b^ | 7.114 | 0.009 **  (0.075) | 1.361 | 0.262  (0.030) | 1.361 | 0.262  (0.030) |
| *Episodic Future Thinking Task* | | | | | | |
| Specificity ^a^ | 0.251 | 0.617  (0.003) | 0.192 | 0.826 (0.004) | 0.594 | 0.555  (0.013) |
| Vividness ^b^ | 9.971 | 0.002 **  (0.102) | 1.405 | 0.251  (0.031) | 1.319 | 0.273  (0.029) |
| Disgust ^b^ | 8.120 | 0.005 **  (0.084) | 0.259 | 0.772  (0.006) | 1.291 | 0.280  (0.029) |

*Notes.* * Significant at the *p* < 0.05 threshold, ** Significant at the *p* < 0.01 threshold. *** Significant at the *p* < 0.001 threshold. ^a^ Researcher rated ^b^ Participant rated. Specificity refers to the proportion of specific autobiographical memories/future events. All analyses were run with age, ethnicity and DASS-Depression entered as covariates.

**Table S6.** Effect of group in non-parametric ANCOVA (Quade’s) model for disgust to future events induced by neutral and negative cues.

| Outcome | F-value  (*df*=2, 114) | *p* value (η2) |
| --- | --- | --- |
| Neutral cue words | 2.692 | 0.072 (0.046) |
| Negative cue words | 3.882 | 0.023 (0.065) *  AN vs HC, *p* = 0.473  BN & BED vs HC, *p* = 0.018 *  AN vs BN & BED, *p* = 0.200 |

*Notes.* All analyses were run with age and ethnicity as covariates. *Significant at p<0.05, **significant at p<0.001. Abbreviations: AN = Anorexia Nervosa; BN&BED = Bulimia Nervosa and Binge Eating Disorders; df = degrees of freedom; HC = Healthy Controls; η^2^ = Partial Eta Squared.

**Table S7.** Results of linear regression models investigating the effect of TQ-R and POBS on autobiographical memory test and episodic future thinking task outcomes in response to negative cues in the healthy control sample (n = 36).

| Tested Model | Dependent Variable | Adjusted R^2^ (SE) | F-value  (*df*=3,32) | *p*-value for overall model | Independent Variable | Unstandardised Beta (SE) | *ß* | t | *p*-value for regressor |
| --- | --- | --- | --- | --- | --- | --- | --- | --- | --- |
| *Autobiographical Memory Task* | | | | | | | | | |
| Model 1 | Specificity^a^ | 0.038 (0.387) | 1.457 | 0.245 | **(Constant)** | 0.934 (0.326) |  | 2.865 | 0.007 * |
|  |  |  |  |  | TQ-R | -0.074 (0.164) | -0.075 | -0.452 | 0.654 |
|  |  |  |  |  | Age | -0.008 (0.011) | -0.116 | -0.678 | 0.503 |
|  |  |  |  |  | Ethnicity | -0.066 (0.032) | -0.353 | -2.057 | 0.048 |
| Model 2 | Vividness^b^ | -0.006 (1.426) | 0.930 | 0.437 | **(Constant)** | 5.286 (1.203) |  | 4.393 | < 0.001 ** |
|  |  |  |  |  | TQ-R | 0.921 (0.603) | 0.260 | 1.526 | 0.137 |
|  |  |  |  |  | Age | -0.030 (0.041) | -0.125 | -0.712 | 0.481 |
|  |  |  |  |  | Ethnicity | -0.038 (0.119) | -0.056 | -0.318 | 0.753 |
| Model 3 | Specificity^a^ | 0.036 (0.387) | 1.432 | 0.252 | **(Constant)** | 0.976 (0.378) |  | 2.585 | 0.014 * |
|  |  |  |  |  | POBS | -0.021 (0.055) | -0.066 | -0.373 | 0.712 |
|  |  |  |  |  | Age | -0.009 (0.012) | -0.144 | -0.791 | 0.435 |
|  |  |  |  |  | Ethnicity | -0.067 (0.033) | -0.356 | -2.060 | 0.048 |
| Model 4 | Vividness^b^ | -0.041 (1.451) | 0.541 | 0.658 | **(Constant)** | 4.885 (1.416) |  | 3.451 | 0.002 * |
|  |  |  |  |  | POBS | 0.225 (0.208) | 0.199 | 1.084 | 0.287 |
|  |  |  |  |  | Age | -0.009 (0.045) | -0.038 | -0.201 | 0.842 |
|  |  |  |  |  | Ethnicity | -0.033 (0.122) | -0.049 | -0.274 | 0.786 |
| *Episodic Future Thinking Task* | | | | | | | | | |
| Model 5 | Specificity^a^ | -0.028 (0.343) | 0.684 | 0.568 | **(Constant)** | 0.170 (0.289) |  | 0.587 | 0.561 |
|  |  |  |  |  | TQ-R | 0.164 (0.145) | 0.195 | 1.133 | 0.266 |
|  |  |  |  |  | Age | 0.005 (0.010) | 0.091 | 0.511 | 0.613 |
|  |  |  |  |  | Ethnicity | -0.012 (0.029) | -0.073 | -0.411 | 0.684 |
| Model 6 | Vividness^b^ | -0.026 (1.371) | 0.707 | 0.555 | **(Constant)** | 3.351 (1.157) |  | 2.898 | 0.007 * |
|  |  |  |  |  | TQ-R | 0.631 (0.580) | 0.187 | 1.088 | 0.285 |
|  |  |  |  |  | Age | 0.017 (0.040) | 0.078 | 0.438 | 0.664 |
|  |  |  |  |  | Ethnicity | -0.068 (0.115) | -0.106 | -0.596 | 0.556 |
| Model 7 | Specificity^a^ | -0.013 (0.341) | 0.846 | 0.479 | **(Constant)** | 0.008 (0.332) |  | 0.024 | 0.981 |
|  |  |  |  |  | POBS | 0.065 (0.049) | 0.240 | 1.325 | 0.194 |
|  |  |  |  |  | Age | 0.011 (0.011) | 0.188 | 1.004 | 0.323 |
|  |  |  |  |  | Ethnicity | -0.009 (0.029) | -0.056 | -0.318 | 0.752 |
| Model 8 | Vividness^b^ | -0.043 (1.383) | 0.523 | 0.669 | **(Constant)** | 3.058 (1.349) |  | 2.267 | 0.030 * |
|  |  |  |  |  | POBS | 0.159 (0.198) | 0.148 | 0.804 | 0.427 |
|  |  |  |  |  | Age | -0.032 (0.043) | 0.142 | 0.747 | 0.461 |
|  |  |  |  |  | Ethnicity | -0.065 (0.116) | -0.100 | -0.558 | 0.51 |

*Notes.* ^a^ Researcher rated, ^b^ Participant rated, *significant at *p*<0.05, **significant at *p*<0.001*.* Specificity refers to the proportion of specific memories/future events. All analyses were run with age, ethnicity (Caucasian vs non-Caucasian), and DASS-Depression scores as covariates. Abbreviations: POBS = The Perception of Betrayal Sensitivity; SE = standard error; TQ-R = Teasing Questionnaire- Revised; *ß* = standardised beta.
